# Field-induced magnetic instability and quantum criticality in the antiferromagnet CeCu_2_Ge_2_

**DOI:** 10.1038/srep18699

**Published:** 2016-01-13

**Authors:** Yi Liu, Donghua Xie, Xiaoying Wang, Kangwei Zhu, Ruilong Yang

**Affiliations:** 1 Science and Technology on Surface Physics and Chemistry Laboratory, P.O.Box 718-35, Mianyang 621907, P.R.China

## Abstract

The magnetic quantum criticality in strongly correlated electron systems has been considered to be closely related with the occurrence of unconventional superconductivity. Control parameters such as magnetic field, pressure or chemical doping are frequently used to externally tune the quantum phase transition for a deeper understanding. Here we report the research of a field-induced quantum phase transition using conventional bulk physical property measurements in the archetypal antiferromagnet CeCu_2_Ge_2_, which becomes superconductive under a pressure of about 10 GPa with Tc ~ 0.64 K. We offer strong evidence that short-range dynamic correlations start appearing above a magnetic field of about 5 T. Our demonstrations of the magnetic instability and the field-induced quantum phase transition are crucial for the quantum criticality, which may open a new route in experimental investigations of the quantum phase transition in heavy-fermion systems.

Superconductivity in heavy-fermion systems is usually found to emerge in the vicinity of a quantum critical point^1^,^2^,^3^,^4^. Intermetallic compounds containing 4f or 5f electrons are prototypical systems to study this novel quantum critical phenomenon, which is controlled by the competing magnetic interaction between single-ion Kondo effect and long-range Rudermann-Kittel-Kasuya-Yoshida (RKKY) exchange interaction^5^,^6^. In Kondo-lattice systems, strong hybridization of the localized f moments with the conduction electrons results in heavy quasiparticles, and the itinerant band electrons screen the localized f electrons, forming a singlet. Weak hybridization, on the other hand, typically yields RKKY-type interactions and magnetically ordered ground states. The competition between Kondo effect and RKKY interaction leads to a quantum critical ground state at zero temperature, which often separates the magnetic ordered state from the weakly interacting paramagnetic state^7^,^8^. The dynamic nature of the quantum critical state, manifested by magnetic fluctuations, is generally probed using external parameters such as pressure, chemical doping, and magnetic field^9^. Although intensive efforts of theoretical and experimental investigations have been focused on the quantum criticality in candidate materials, there are still important questions to be addressed in order to develop an unambiguous formulation.

The compound CeCu_2_Ge_2_ is an archetypal metal where correlated Ce^3+^ ions ordered antiferromagnetically below T_N_ ≈ 4 K^ ^10. It belongs to CeM_2_T_2_ family, crystallizing in ThCr_2_Si_2_-type tetragonal structure (space group I4/mmm), where M is a transition metal element and T is Si or Ge^11^. When a pressure of about 10 GPa is applied, CeCu_2_Ge_2_ exhibits unconventional superconductivity with T_C_ ≈ 0.64 K^12^. Recently, the compound CeCu_2_Ge_2_ was shown to undergo a quantum phase transition in applied magnetic field of about 8 T as temperature approaches zero, and the microscopic origin was dominated by fluctuations of the long-range order parameter, as envisaged on Hertz–Millis–Moriya spin-fluctuation theory^13^,^14^,^15^,^16^,^17^. Detailed neutron scattering measurements were performed as a function of field or temperature to understand the development of the quantum criticality^13^,^18^. However, the understanding is incomplete and inconclusive. In this work, we study the quantum phase transition of the compound CeCu_2_Ge_2_ in applied magnetic fields using bulk physical property measurements like magnetization, AC magnetic susceptibility, and resistivity. Strong evidence of short-range dynamic correlations starts appearing within fields in excess of 5 T. Interestingly, the field-induced magnetic instability and the associated quantum phase transition in CeCu_2_Ge_2_ certainly shed new light on the quantum criticality in heavy-fermion systems.

## Results

### Magnetization measurements

**Figure 1:**
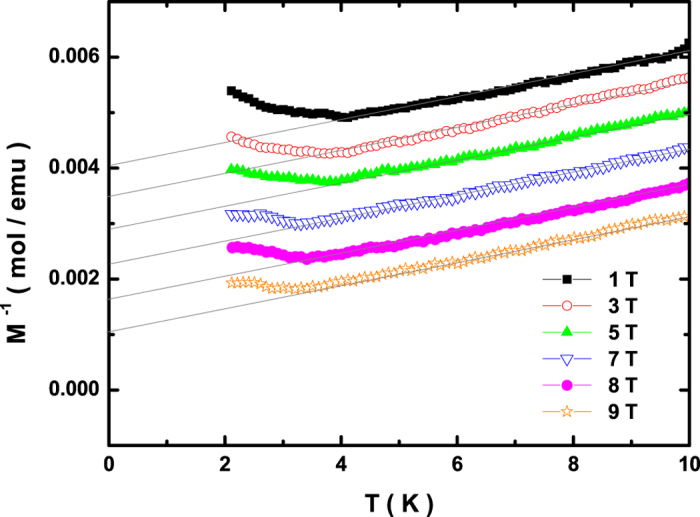
Low-temperature bulk inverse magnetization of the antiferromagnet CeCu_2_Ge_2_ measured at different magnetic fields. The inverse magnetization plots at different fields are vertically offset by finite values for clarity.

Figure 1 shows bulk inverse magnetization of the antiferromagnet CeCu_2_Ge_2_ at low temperatures. The inverse magnetization data recorded under different magnetic fields are respectively plotted with vertical offset of finite values for clarity. In the high-temperature paramagnetic region, all inverse magnetization shows linear temperature dependence, following the Curie-Weiss (CW) law^5^,^19^. With decreasing temperature a sharp upturn denoted as T_N_ is observed in the inverse magnetization, and the deviation from the CW law indicates the appearance of long-range antiferromagnetic order. As extracted from the magnetization data, the antiferromagnetic transition located exactly at 4.2 K is suppressed to be at 3.5 K in a magnetic field of 9 T. Additionally, there exists another small kink at about 2.8 K in the inverse magnetization, weakening the upturn of the inverse magnetization at high fields. This anomaly is previously reported to involve a field-induced quantum phase transition by neutron scattering measurements^18^.

### AC magnetic susceptibility measurements

**Figure 2:**
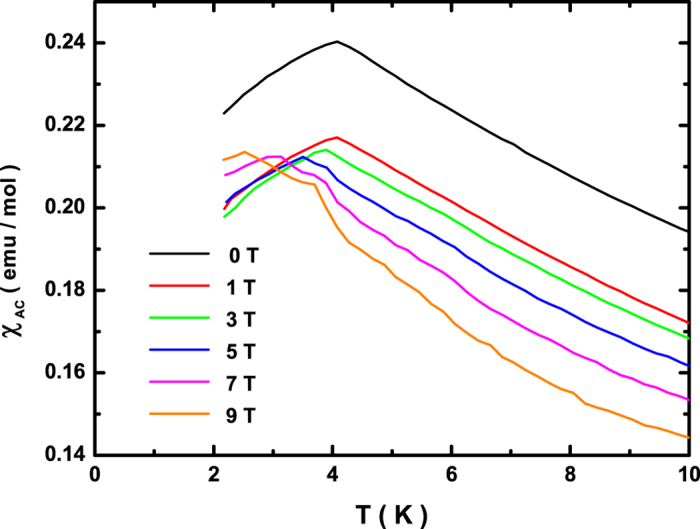
Low-temperature bulk AC magnetic susceptibility of the antiferromagnet CeCu_2_Ge_2_ measured under different DC bias fields. The amplitude of the AC excitation field is 10 Oe, and the test frequency is 9999 Hz.

To illustrate the quantum phase transition, we have measured the low-temperature bulk AC magnetic susceptibility of the antiferromagnet CeCu_2_Ge_2_ under different DC bias fields, as shown in Fig. 2. The AC susceptibility data is measured with an AC excitation field of 10 Oe operating at 9999 Hz. At zero field, a peak-like anomaly in the AC susceptibility at about 4 K is featured as the antiferromagnetic transition. When DC bias magnetic fields are applied, the AC susceptibility is markedly suppressed. Nevertheless, except the antiferromagnetic transition, the AC susceptibility at extremely low temperatures exhibits another peak-like anomaly above 5 T, implying the field-induced quantum phase transition. The enhanced magnitude of the AC susceptibility fades rapidly at elevated temperatures, signifying the quantum nature of the dynamic correlations.

**Figure 3:**
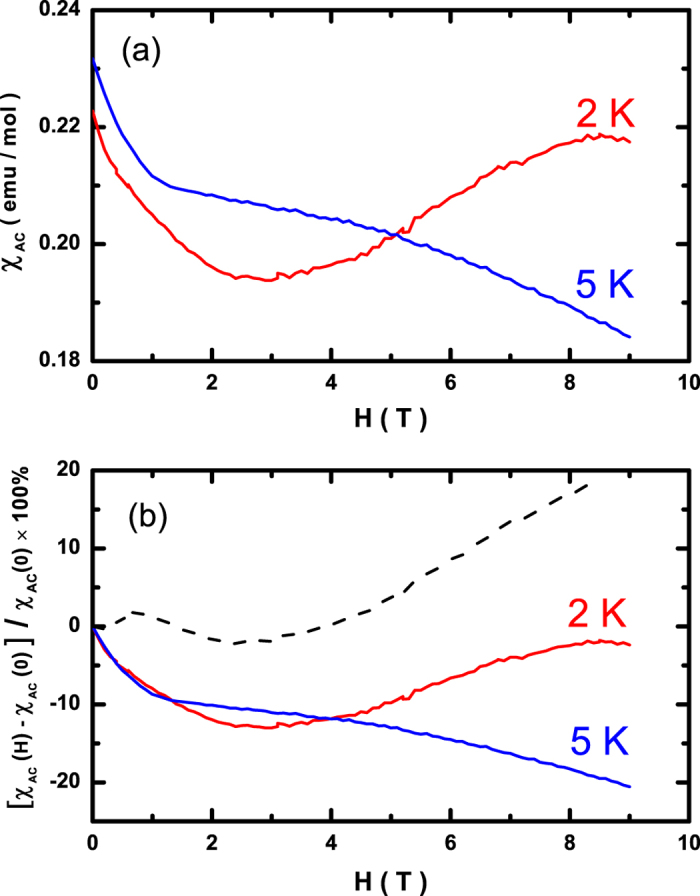
Field dependence of the bulk AC magnetic susceptibility of the antiferromagnet CeCu_2_Ge_2_ measured at specified temperatures 2 K and 5 K. (**a**) AC susceptibility data χ_AC_. (**b**) Renormalization of χ_AC_ using formula [χ_AC_ (H) − χ_AC_ (0)]/χ_AC_ (0) × 100%. Dashed line indicates the difference between the two curves.

We have also measured the AC susceptibility as a function of magnetic field to unambiguously establish the field-induced quantum phase transition. As shown in Fig. 3(a), the AC susceptibility at 2 K first decreases rapidly at low fields, followed by a slow increase above 5 T, finally yielding a shoulder-like plateau at around 8 T. The exotic behavior of the AC susceptibility data clearly reveals the existence of the field-induced quantum phase transition. The AC susceptibility at 5 K (in the paramagnetic state) is likewise plotted in Fig. 3(a) for comparison, which shows a monotonic decrease with increasing fields. In Fig. 3(b), the AC susceptibility data are renormalized using formula [χ_AC_ (H) − χ_AC_ (0)]/χ_AC_ (0) × 100% for clarity, and the dashed line indicates the difference between the two curves, showing the tendency of the divergence at a higher field.

### Resistivity measurements

**Figure 4:**
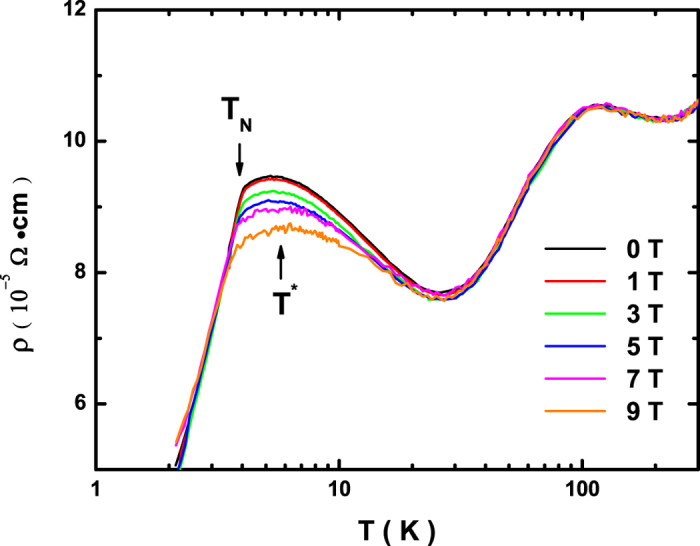
Bulk resistivity of the antiferromagnet CeCu_2_Ge_2_ measured under different magnetic fields.

We now turn to resistivity measurements for help in understanding of the underlying mechanisms. Figure 4 shows the temperature dependence of the bulk resistivity of the antiferromagnet CeCu_2_Ge_2_ under various DC bias magnetic fields. Magnetic fields are applied parallel to the direction of the test current. In all the resistivity curves, two prominent features, which are typical to heavy-fermion systems, have been observed. The broad peak around 100 K is caused by crystal-field splitting, and the other peak at 6 K gives evidence for the Kondo-lattice coherence temperature T^*^, which corresponds to the formation of heavy quasiparticles^20^. At lower temperatures below T_N_ ≈ 4 K, the resistivity drops rapidly. In the magnetoresistance measurements, applying external magnetic fields complicates the low-temperature resistivity behavior^21^. The bulged peak at T^*^ is gradually suppressed at high fields, manifested by a negative magnetoresistance effect, and simultaneously a positive magnetoresistance below T_N_ is observed. The negative magnetoresistance at T^*^ (about −10% at 9 T) is likely to be associated with the suppression of local Kondo interactions. We note that T^*^ is very close to T_N_, so this negative magnetoresistance cannot exclude contributions from the suppression of T_N_ or spin fluctuations.

## Discussion

In the quantum criticality scenario, our experimental investigations clearly show that strong evidence of short-range dynamic correlations starts appearing above 5 T, revealing the existence of the field-induced quantum phase transition by using conventional bulk physical property measurements. In the antiferromagnet CeCu_2_Ge_2_, the disappearance of T_N_ by externally applying pressure or magnetic field is accompanied by extremely strong quantum fluctuations. At a generic quantum critical point, the Gruneisen ratio Γ diverges, and when scaling applies, Γ ~ T^−1/(vz)^ at the critical point P = P_C_ or H = H_C_, providing a means to measure the scaling dimension^22^,^23^. In the limit T → 0 and P≠P_C_ or H≠H_C_, Γ ~ 1/(P-P_C_) or 1/(H-H_C_) with a prefactor that is a simple combination of critical exponents. These theoretical predictions are phenomenally in consistence with our results in CeCu_2_Ge_2_.

The critical field of about 8 T for the quantum phase transition was first identified by neutron studies^13^. However, this conclusion is elusive, because T_N_ persists at 14 T and is even estimated to vanish at 31–35 T by using quadratic fitting^21^. As a consequence, the 8 T field could not be simply considered as the quantum critical point, confirming the complexity of the magnetic phase diagram in CeCu_2_Ge_2_. In recent high-field resistivity and torque magnetometry measurements, the quantum phase transition for H ≈ 8 T applied along [−110] direction is suggested to be a first-order metamagnetic transition when H ≈ 10 T is applied along [100] direction^24^. This first-order transition corresponds to a first-order line in the T-H diagram, and ends at an end point at higher temperatures where it becomes strictly second order in nature. As discussed in Ref. 24, the first-order transition disappears when H is tilted towards the c axis, implying that the end point must move to T = 0 K at a certain angle, where the quantum critical end point can be accessed by varying the field orientation with respect to the crystallographic axes. In our work, since all the measurements were performed on polycrystalline samples, the field-induced effects behave as an average from different directions of the single crystal. In other words, only parts of the polycrystalline samples were tuned to the quantum critical end point by external magnetic field. In this manner, the exotic behavior near 8 T in magnetization and AC susceptibility measurements arises from the mixture or the competition of thermal and quantum fluctuations. In the same way, what we really observe looks more like some crossover behaviors, and for the time being the class of the quantum critical point can not be simply determined, which deserve further studies to fully demonstrate the quantum criticality.

As the archetypal antiferromagnet CeCu_2_Ge_2_ is superconductive under ~10 GPa pressure, our demonstrations of the field-induced quantum phase transition certainly enrich the understanding of the quantum criticality in the ideal system CeCu_2_Ge_2_ as well as other heavy-fermion systems. In actual fact, most present available experiments utilize magnetic fields which are not enough to study the quantum criticality, and the system still resides in the magnetically ordered region. Thus, more high-field experiments are needed to really cover the field-induced quantum phase transition. Moreover, high-pressure accessing techniques used to encounter enormous difficulties, hindering the complete understanding of the quantum phase transition by neutron scattering measurements^13^. And herein the bulk physical property measurements used in this work can be easily combined with high-pressure accessions, providing a new route in experimental investigations of the quantum criticality.

## Methods

Measurements were performed on polycrystalline CeCu_2_Ge_2_ samples, which were cut from an ingot prepared by arc melting of stoichiometric 99.9% cerium, 99.99% copper, and 99.999% germanium. The phase of this intermetallic was confirmed by X-ray diffractions, and no impurities were detected. Magnetization and AC susceptibility measurements were performed at ambient pressure using a Quantum Design physical property measurement system. Resistivity was measured using a standard four-wire method with an AC excitation operating at 17 Hz. Magnetoresistivity measurements were performed with applied various DC magnetic fields which are parallel to the direction of the test current.

## Additional Information

**How to cite this article**: Liu, Y. *et al.* Field-induced magnetic instability and quantum criticality in the antiferromagnet CeCu_2_Ge_2_. *Sci. Rep.* **6**, 18699; doi: 10.1038/srep18699 (2016).
